# Use of Data Mining to Determine Usage Patterns of an Online Evaluation Platform During the COVID-19 Pandemic

**DOI:** 10.3389/fpsyg.2020.588843

**Published:** 2020-09-25

**Authors:** Rafael E. Reigal, José Luis Pastrana-Brincones, Sergio Luis González-Ruiz, Antonio Hernández-Mendo, Juan Pablo Morillo-Baro, Verónica Morales-Sánchez

**Affiliations:** ^1^University of Málaga, Málaga, Spain; ^2^Department of Languages and Computer Science, University of Málaga, Málaga, Spain; ^3^Department of Social Psychology, Social Work, Anthropology and East Asian Studies, University of Málaga, Málaga, Spain

**Keywords:** menpas, COVID-19, data mining, clustering, human behavior

## Abstract

MenPas is a psychosocial assessment platform^1^ developed by the University of Malaga in 2008. There has been a significant increase in data traffic during the period of confinement by COVID-19 (March and April ’20) compared to the same period in the previous year. The main goal to achieve in this work is to determine the patterns of use of this platform on both period of time. So, we want to respond to the following question: So, we the following question: Has the COVID-19 Pandemic changed the pattern of the Menpas users? In order to respond it, cluster analysis techniques (Data Mining) have been used to classify people taking surveys into quotient sets (cluster). This is a multivariate technique for dividing data into sets to that are as homogeneous as possible within themselves and heterogeneous among themselves. Specifically, the K-Means algorithm has been used for this analysis, which is based on the evaluation of the distance between data and the average of each variable. So, it is recommended to discover patterns or relationships among the data. Specifically, the use of the following questionnaires has been analyzed: Competitive State Anxiety Inventory-2 (CSAI-2), State Trait Anxiety Inventory (STAI), Profile of Mood State (POMS), Resilience Scale (RS), Sport Performance Psychological Inventory (IPED), Maslach Burnout Inventory (MBI) and Self-concept Form-5 (AF-5). The analyses have shown changes in cluster formation between 2019 and 2020 based on the variables gender, age, marital status or physical practice. Therefore, the analyses carried out have been sensitive to determine several profiles of people using the MenPas platform because there are changes in the characteristics of the user groups that have carried out the analyzed tests.

## Introduction

SARS-CoV-2 (coronavirus 2019, COVID-19) has generated an exceptional situation (López and Rodó, 2020) in this year 2020. Although many other viruses had appeared years ago, that had not generated an emergency as globalized as it has happened this time (Sohrabi et al., 2020). This virus, originated in China according to official sources, has been spreading rapidly throughout the world. The World Health Organization (WHO) declared the pandemic on March 11, and a large number of countries have had to establish measures to cope with the spread of infections (Chinazzi et al., 2020; Parmet and Sinha, 2020; Studdert and Hall, 2020). For example, Spain declared the state of alarm on March 14th, 2020, by Royal Decree and published in the Official State Bulletin, establishing restrictive measures for mobility and limiting the activities that could be carried out.

This recent unprecedented situation has caused changes in people’s habits and practices, modifying the way of living before. For example, educational centers have been closed, non-essential productive activities have had to stop, sports competitions have been canceled, or household supplies of basic consumer products have been carried out following strict security measures (e.g., Corsini et al., 2020; Viner et al., 2020; Zhang et al., 2020). This has forced to people modifying habitual routines and adapting to this new situation, which has meant reducing social contact, using new strategies to communicate with family and friends, modifying physical activity or feeding habits, as well as the way of studying and working (e.g., Basilaia and Kvavadze, 2020; Belzunegui-Eraso and Erro-Garcés, 2020; Butler and Barrientos, 2020; Martínez-Ferrán et al., 2020).

Along these months, the available technology has facilitated communication helping for educational or work activities to be carried out (Ting et al., 2020). Online platforms have been a useful alternative way for students to continue learning or for many companies to continue operating (Bao, 2020; Basilaia and Kvavadze, 2020). The use of new information and communication technologies is already a settled reality in many areas, but this period has meant an acceleration in the technological transition that will possibly mark a before and after in the use of this type of software (Belzunegui-Eraso and Erro-Garcés, 2020; Dong et al., 2020; Kousha and Thelwall, 2020).

The online evaluation platform MenPas (see footnote 1) (González-Ruiz et al., 2010, 2018) is a virtual platform that brings together a wide set of tools that are used for psychosocial assessment. Its use is very widespread along different countries and the number of users has been increased in recent years. MenPas has been used in multiple investigations to gather study data, which can be verified in the published literature (e.g., Aragón et al., 2017; Reigal et al., 2019; González-Guirval et al., 2020). Through this platform, groups of people in investigations can be managed and data can be gathered for further processing. It is an online platform with easy access, programed to be used from multiple devices, which facilitates its implementation.

**FIGURE 1:**
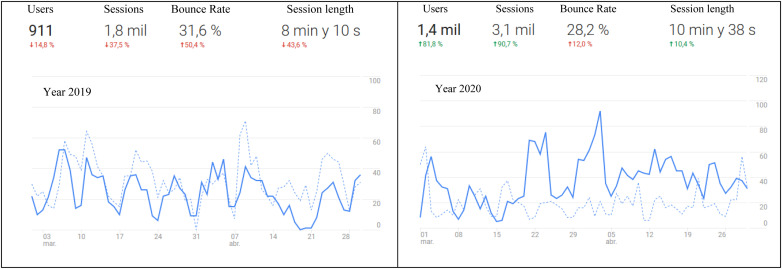
MenPas users (March and April ’19 vs. March and April ’20) by Google analytic.

**FIGURE 2:**
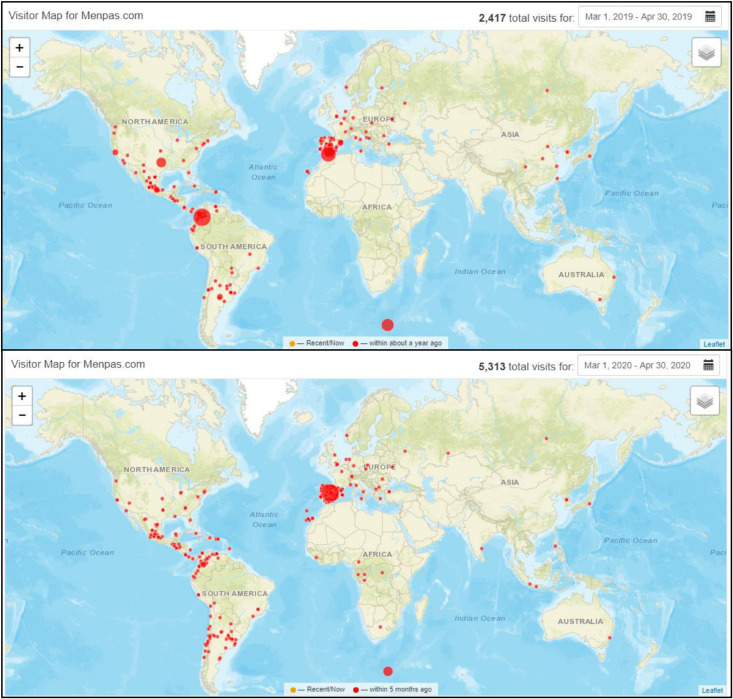
MenPas users (March and April ’19 vs. March and April ’20) by ClustrMaps.

The large amount of data that has been gathered in recent years by MenPas, allows us to obtain a lot of information about the type of user that connects to the platform, as well as the tools that are mostly used. In addition to knowing those aspects that generate the most interest among users, the global analysis of the data can provide information on population trends. That is, if there are changes in the needs, interests or habits of people. The pandemic declared by the WHO and the restrictions in mobility established in some countries have altered people’s habits and have affected specific aspects of their mental health (Banerjee and Rai, 2020; Duan and Zhu, 2020; Li et al., 2020). Global usage data from March to May 2020 compared to the same period in 2019, shows that there has been a high increase in the number of MenPas users. The platform’s internal event control, the associated Google Analytic account^2^ (1800 vs. 3100 sessions) (Figure 1) and the added widget of ClustrMaps^3^ (2400 vs. 5300 visits) (Figure 2) show clear information about this fact.

Specific online resources have had to be used to study and work, and people have had to change their way of living with others and his way of understanding his environment (Torales et al., 2020; Wang G. et al., 2020; Wang C. et al., 2020). The lack of studies that provide information on changes in uses and customs through the analysis of online platforms, joined to the fact that currently virtual platforms have significantly increased their use, provide essential information for understanding changes in the population’s behavior. Therefore, platforms such as MenPas can help to understand these changes by analyzing their use. The main goal of this study is to get a significant knowledge about patterns in the use of on-line platforms when a socio-economic and environmental situation changes. These patterns could show how the members of a society adapt their behavior and find a way to adapt and achieve their needs in that situation. So, the main objective was to determine if the COVID-19 Pandemic has changed the pattern of Menpas users. In order to determine this, the use of the following questionnaires was specifically explored: Competitive State Anxiety Inventory-2 (CSAI-2), State Trait Anxiety Inventory (STAI), Profile of Mood State (POMS), Resilience Scale (RS), Sport Performance Psychological Inventory (IPED), Maslach Burnout Inventory (MBI), and Self-concept Form-5 (AF-5). Two different anxiety questionnaires have been used because both are available at the platform and they consider different aspects on anxiety evaluation: CSAI-2 tests anxiety in sport and STAI tests general anxiety. The information from the following variables have been gathered each time a questionnaire has been taken by one user: his/her age, his/her gender, the sport played, how many hours has been playing that sport, his/her marital status, his/her educational level, his/her profession, the questionnaire taken and the date when it was taken.

## Materials and Methods

### Participants

**FIGURE 3:**
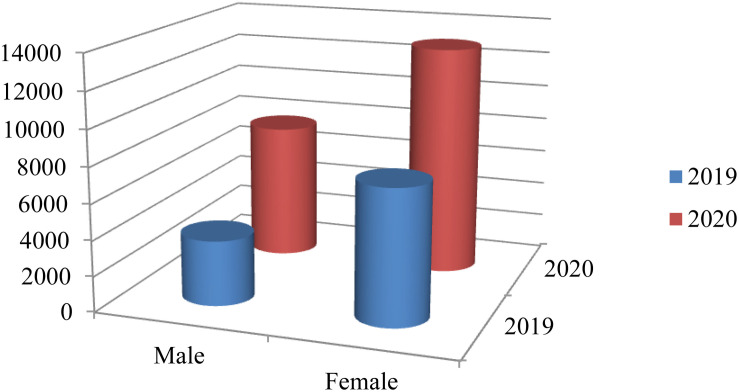
Participants.

As shown in Figure 3, we have collected 11263 records from March the 1st to April the 30th in 2019 (Male: 3687 - 32,74% and Female: 7576 - 67,26%) and 20627 records from March the 1st to April the 30th in 2020 (Male: 7655 - 37,11% and Female: 12972 - 62,89%) of people who took any of the following questionnaires: Competitive State Anxiety Inventory-2 (CSAI-2), State Trait Anxiety Inventory (STAI), Profile of Mood State (POMS), Resilence Scale (RS), Sport Performance Psychological Inventory (IPED), Maslach Burnout Inventory (MBI) and Self-concept Form-5 (AF-5) in MenPas (see footnote 1). Menpas is an on-line software platform for psychosocial assessment (González-Ruiz et al., 2010, 2018). This study has been performed using data from 803 countries over the world (see footnote 3) where users are mainly from Spain, Colombia, United States, Mexico, Argentine, Guatemala, Chile, Costa Rica, El Salvador, Portugal, China, Ecuador, Russia, Peru, United Kingdom, Republic of Korea, Brazil, and Cuba.

### Instruments

(a) Competitive State Anxiety Inventory-2 (CSAI-2, Martens et al., 1990). This questionnaire allows evaluating anxiety competitive. It is made up of 27 items, which are structured in three factors: cognitive anxiety, somatic anxiety and self-confidence. This instrument is answered using a Likert-type scale from 1 (almost never) to 5 (almost always).
(b) State Trait Anxiety Inventory (STAI, Spielberg et al., 1970). This questionnaire allows evaluating state and trait anxiety. It consists of 40 items and two factors: state anxiety and trait anxiety. Scores in each can range from 0 to 60 points. Each item is answered based on 4 levels 0, 1, 2, and 3).
(c) Profile of Mood State (POMS, McNair et al., 1971). This questionnaire evaluates mood states. It is made up of 65 items and evaluates seven dimensions: tension, depression, anger, vigor, fatigue, confusion and friendship. The answers are answered on a scale from 0 (not at all) to 4 (very much)].
(d) Resilence Scale (RS, Wagnild and Young, 1993). This scale assesses the level of individual resilience, that is, the ability to resist various stressors and adapt to life before them. It is made up of 25 items and two factors: personal competence and acceptance of oneself and of life. This instrument is answered using a Likert-type scale from 1 (disagree) to 7 (totally agree).
(e) Sport Performance Psychological Inventory (IPED, Hernández-Mendo, 2006; Hernández-Mendo et al., 2014) is a Spanish adaptation of the Psychological Performance Inventory (PPI) (Loehr, 1986, 1990). It is used to evaluate several psychological skills used by athletes during competition. It consists of 42 items, divided into the following dimensions: self-confidence, negative coping control, attention control, visual-imagery control, motivational level, positive coping control, and attitude control. This instrument is answered using a Likert-type scale from 1 (almost never) to 5 (almost always).
(f) Maslach Burnout Inventory (MBI, Maslach and Jackson, 1981). This scale assesses the level of burnout at work. It is made up of 22 items and three factors: emotional exhaustion, depersonalization and lack of personal accomplishment. This instrument is answered using a Likert-type scale from 0 (never) to 6 (everyday).
(g) Self-concept Form-5 (AF-5, García and Musitu, 2001). This questionnaire evaluates the multidimensional self-concept. It is made up of 30 items and evaluates the following dimensions: academic/work, social, emotional, family and physical. Responds with a response scale with values from 1 (totally disagree) and 99 (totally agree).

### Procedure

Data Mining clustering techniques have been used to classify people taking surveys into quotient sets (cluster). Clustering is an unsupervised Machine learning technique for automatic grouping of data whose most popular clustering algorithm is the K-Means. This is a multivariate technique for dividing data into sets to that are as homogeneous as possible within themselves and heterogeneous among themselves. Specifically, the K-Means algorithm has been used for this analysis, which is based on the evaluation of the distance between data and the average of each variable. So, it is recommended to discover patterns or relationships among the data. Specifically, the use of the following questionnaires has been analyzed: Competitive State Anxiety Inventory-2 (CSAI-2), State Trait Anxiety Inventory (STAI), Profile of Mood State (POMS), Resilence Scale (RS), Sport Performance Psychological Inventory (IPED), Burnout and Self-concept Form-5 (AF-5).

**FIGURE 4:**
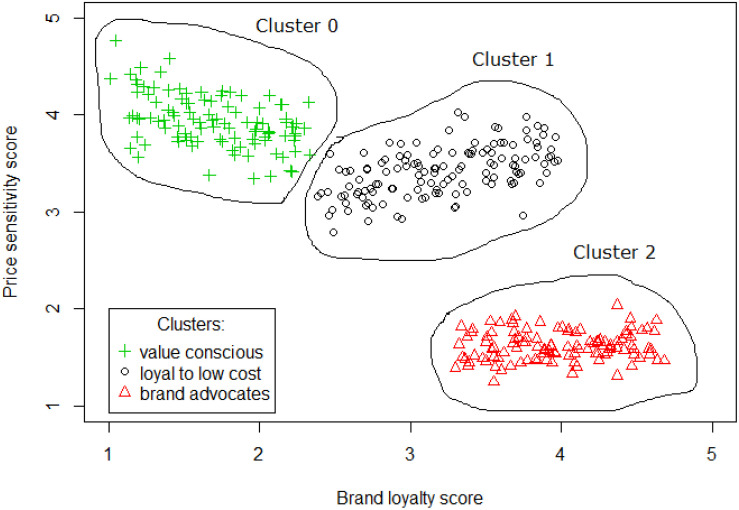
Customers clustering based on their sensitivity to price and brand loyalty.

Figure 4 (got from https://select-statistics.co.uk/blog/customer-segmentation/) shows an example where a group of customers have been segmented based on their sensitivity to price and brand loyalty.

The most widely used clustering algorithm is K-Means because it has a very good scalability with the amount of data. The main problem for using K-Means is that you must specify the number of groups you want to find. This number of groups is called K.

K-Means algorithm follows these steps:

1. Initialization: the location of the centroids of the K groups is chosen at random.
2. Assignment: each data item is assigned to the nearest centroid.
3. Update: the centroid position is updated to the arithmetic mean of the items in the data assigned to the group.

Steps 2 and 3 are followed iteratively until there are no more changes.

### Data Analysis

Following questionnaires have been analyzed: Competitive State Anxiety Inventory-2 (CSAI-2), State Trait Anxiety Inventory (STAI), Profile of Mood State (POMS), Resilence Scale (RS), Sport Performance Psychological Inventory (IPED), Burnout and Self-concept Form-5 (AF-5).

We have made two clustering analyses for each type of questionnaire: 3 and 5 cluster analyses for 2019 and 2020. It has been taken in account the following attributes (variables): age, gender, sport, hours doing sport, marital status, educational level, profession and date.

In addition, a decision tree has been built to respond to the following question in 2019 and 2020: “What is the profession of those who have taken the test in terms of gender, age, hours spent doing sports, marital status and date of the questionnaire?”

## Results

Here we will show the results got grouped by questionnaire. As we told before, we will make the March to May 2019 versus 2020 comparison taking 3 and 5 cluster. So, we will get the 3 and the 5 most representative people taking the survey for each type of questionnaire in 2019 and 2020 and we will compare the main differences on their characteristics based on age, gender, sport, hours doing sport, marital status, educational level, profession and date they took the survey. The effect produced by selecting 5 clusters has been that the bigger groups have been split but keeping the same characteristics, so we will only present the 3 clusters table of results but 5 clusters results are available by any request.

In addition, we are also building a decision tree for each questionnaire to respond to the following question in 2019 and 2020: “What is the profession of those who have taken the test in terms of gender, age, hours spent doing sports, marital status and date of the questionnaire?”

### Competitive State Anxiety Inventory-2 (CSAI-2)

Table 1 shows the 3 clusters got for CSAI-2 questionnaire in 2019 and Table 2 shows the 2020 results. Red columns represent the less significate cluster attending to the number of elements belonging to the cluster and green columns represent the most significate one. Each cluster also shows the number of people belonging to it and the per cent it represents.

**TABLE 1:**
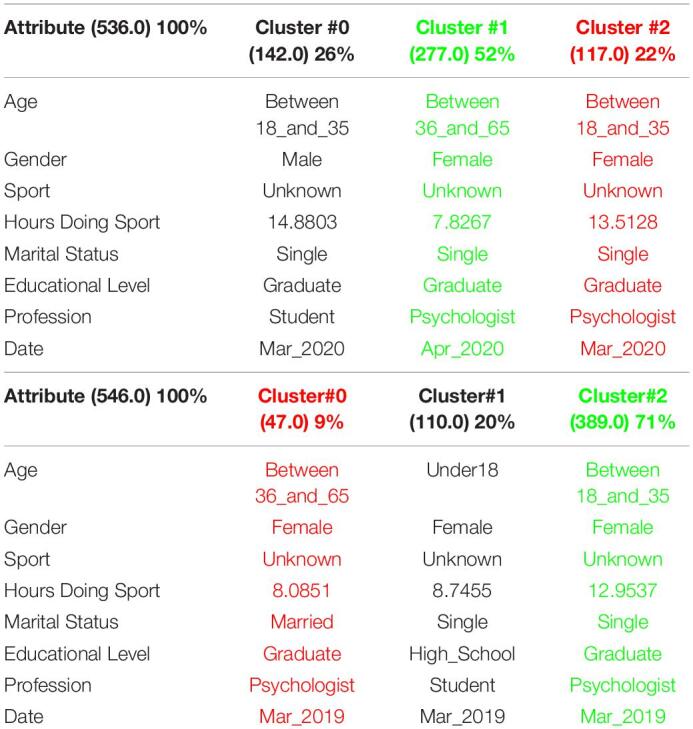
3 clusters got for CSAI-2 questionnaire in 2019 and CSAI-2 analysis for March to May 2019.

**TABLE 2:**
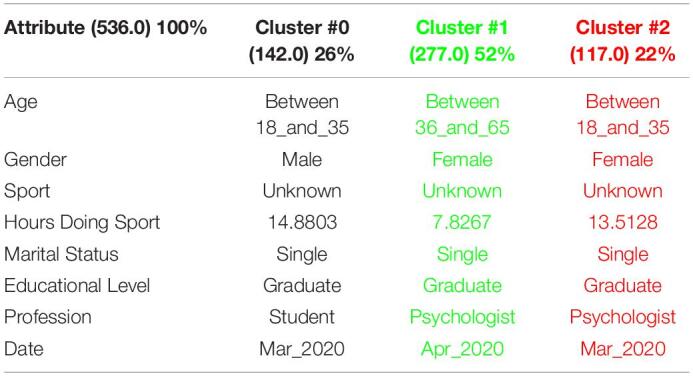
3 clusters CSAI-2 analysis for March to May 2020.

It can be seen that 2020 groups are more homogeneous than 2019. Although they both mainly are women, single, graduates, psychologists, the predominant age in 2020 has gone from the interval 18-35 to the interval 36-65.

Another data of special interest lies in the Gender. While the number of men is not representative to be able to form a group in 2019, the increase of men who carry out the questionnaire in 2020 leads to the creation of a group of men who represent almost a quarter of the population studied. It is also significate how the number of hours doing sports has been increased during the quarantine.

**FIGURE 5:**
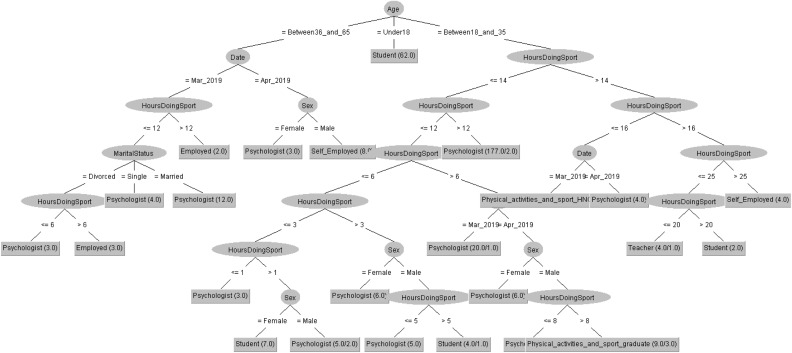
Profession of people taking the CSAI-2 questionnaire in 2019 decision tree.

**FIGURE 6:**
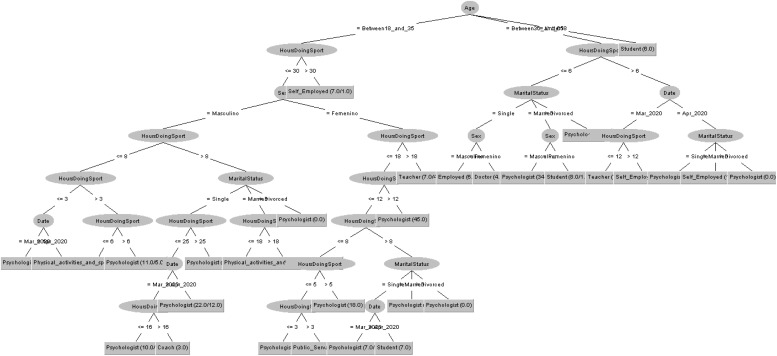
Profession of people taking the CSAI-2 questionnaire in 2020 decision tree.

In order to respond to the question raised before, Figures 5, 6 decision trees shows that most of people taking the CASI-2 questionnaire are Psychologist.

### State Trait Anxiety Inventory (STAI)

Table 3 shows the 3 clusters got for STAI questionnaire in 2019 and Table 4 shows the 2020 results. Red columns represent the less significate cluster attending to the number of elements belonging to the cluster and green columns represent the most significate one. Each cluster also shows the number of people belonging to it and the per cent it represents.

**TABLE 3:**
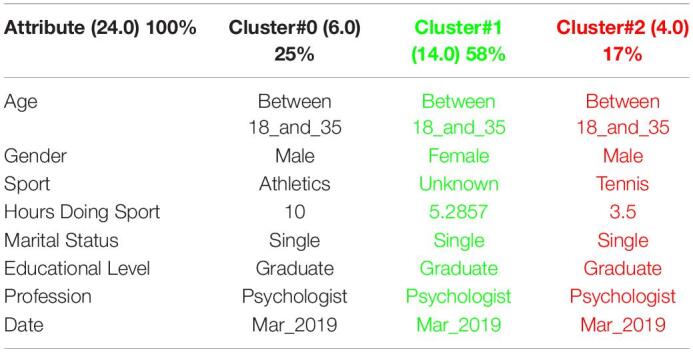
3 clusters STAI analysis for March to May 2019.

**TABLE 4:**
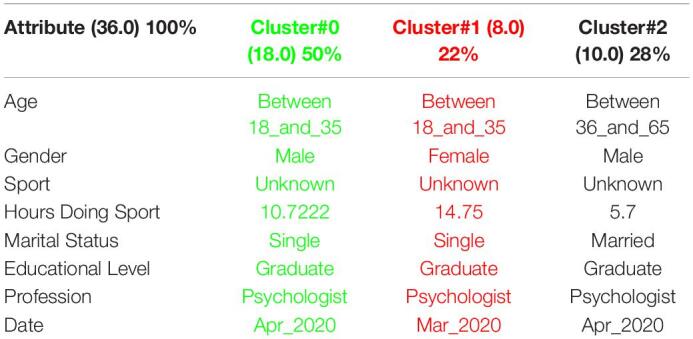
3 clusters STAI analysis for March to May 2020.

Results got in this questionnaire are similar to results got in CSAI-2. This is a coherent result because both of them are related to anxiety. It can be seen how the number of men has been increased in 2020 as well as the age has done, so older people took the anxiety questionnaires in the quarantine.

**FIGURE 7:**
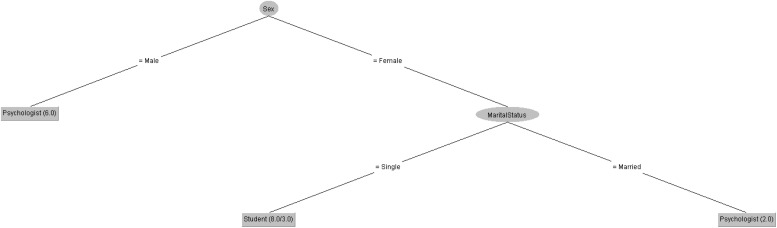
Profession of people taking the STAI questionnaire in 2019 decision tree.

**FIGURE 8:**
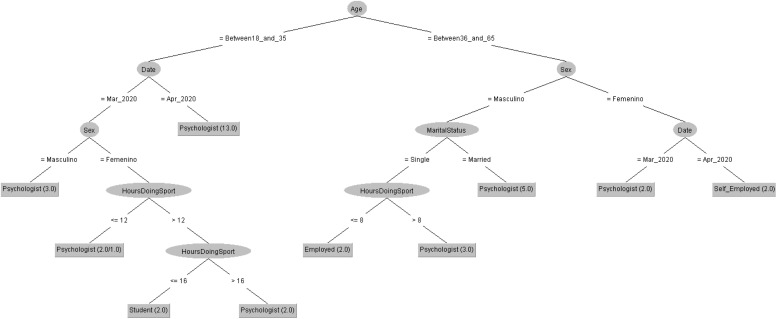
Profession of people taking the STAI questionnaire in 2020 decision tree.

In order to respond to the question raised before, Figures 7, 8 decision trees shows that most of people taking the CASI-2 questionnaire are also Psychologist.

### Profile of Mood State (POMS)

Table 5 shows the 3 clusters got for POMS questionnaire in 2019 and Table 6 shows the 2020 results. Red columns represent the less significate cluster attending to the number of elements belonging to the cluster and green columns represent the most significate one. Each cluster also shows the number of people belonging to it and the per cent it represents.

**TABLE 5:**
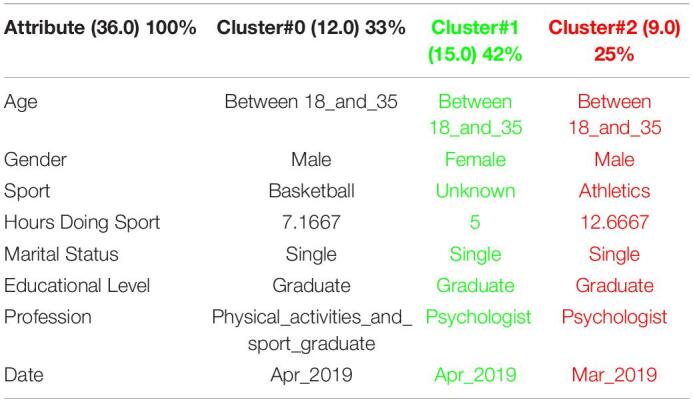
3 clusters POMS analysis for March to May 2019.

**TABLE 6:**
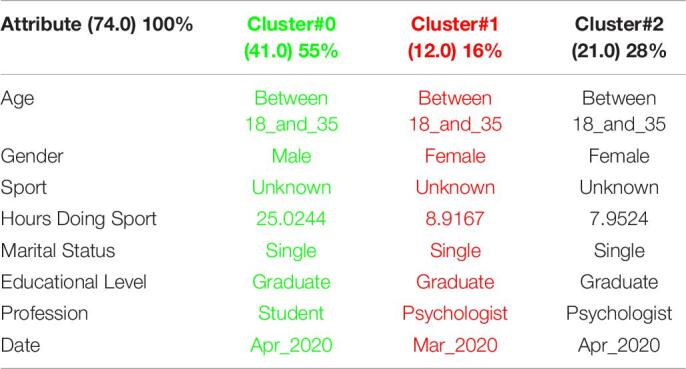
3 clusters POMS analysis for March to May 2020.

Results got shows that there is a new type of person taking the POMS survey in 2020 that was not significate in 2019. It is a man between 18 and 35 years old, student and doing sport 25 h per week what it means a 100% more than 2019.

**FIGURE 9:**
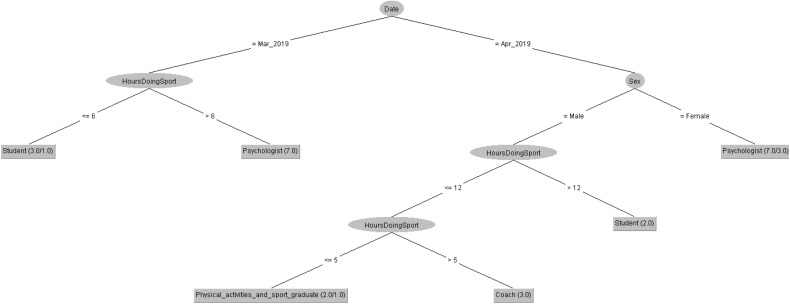
Profession of people taking the POMS questionnaire in 2019 decision tree.

**FIGURE 10:**
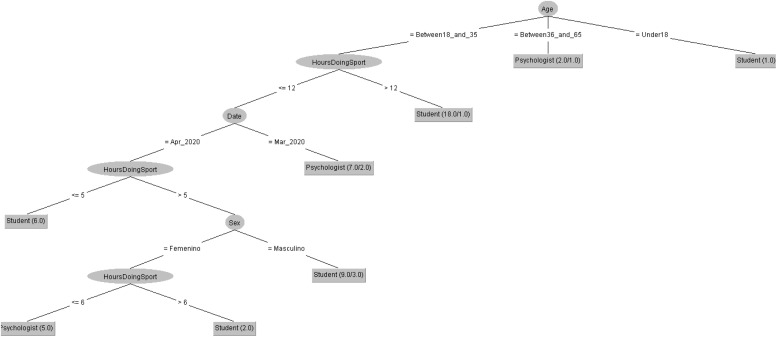
Profession of people taking the POMS questionnaire in 2020 decision tree.

In order to respond to the question raised before, Figures 9, 10 decision trees shows that most of people taking the POMS questionnaire are either Psychologist or students. However, the profession is mostly influenced by the number of hours doing sport in 2019 but by the age in 2020.

### Resilence Scale (RS)

Table 7 shows the 3 clusters got for RS questionnaire in 2019 and Table 8 shows the 2020 results. Red columns represent the less significate cluster attending to the number of elements belonging to the cluster and green columns represent the most significate one. Each cluster also shows the number of people belonging to it and the per cent it represents.

**TABLE 7:**
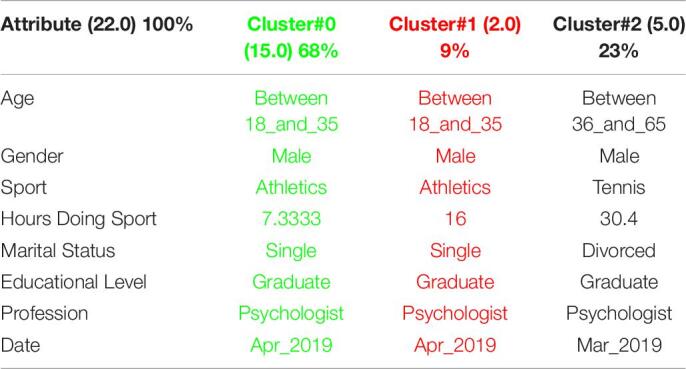
3 clusters RS analysis for March to May 2019.

**TABLE 8:**
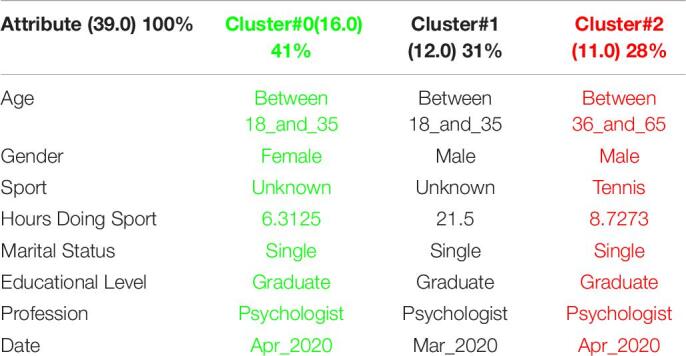
3 clusters RS analysis for March to May 2020.

One of the most significate facts in RS results is that the typical person taking the survey in 2019 was a man but in 2020 there was as male as female. It is also interesting that there was a cluster made by Divorced in 2019 but in 2020 every cluster represents to single people.

**FIGURE 11:**
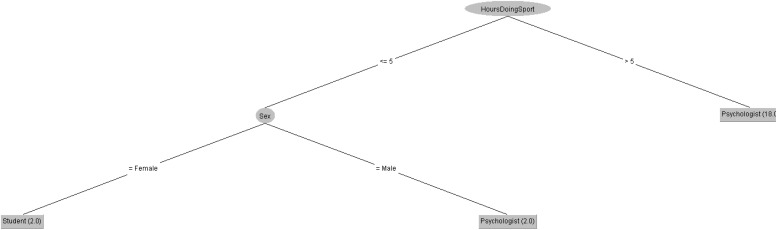
Profession of people taking the RS questionnaire in 2019 decision tree.

**FIGURE 12:**
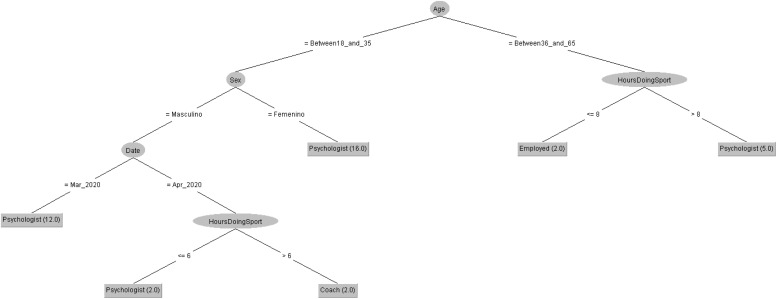
Profession of people taking the RS questionnaire in 2020 decision tree.

In order to respond to the question raised before, Figures 11, 12 decision trees shows that most of people taking the RS questionnaire are either Psychologist or students in 2019 depending on gender and hours doing sport. However, the profession is more heterogeneous in 2020 and the age is one of the most important points for the decision.

### Sport Performance Psychological Inventory (IPED)

Table 9 shows the 3 clusters got for IPED questionnaire in 2019 and Table 10 shows the 2020 results. Red columns represent the less significate cluster attending to the number of elements belonging to the cluster and green columns represent the most significate one. Each cluster also shows the number of people belonging to it and the per cent it represents.

**TABLE 9:**
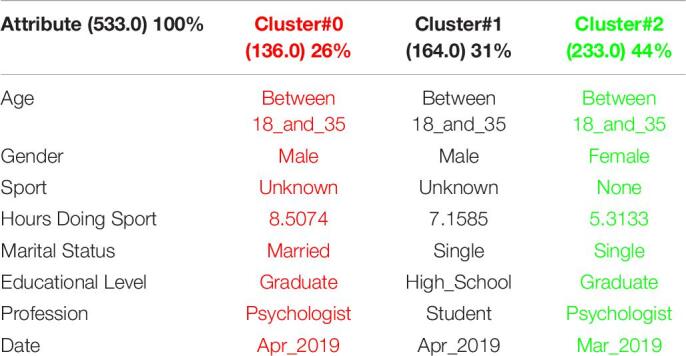
3 clusters IPED analysis for March to May 2019.

**TABLE 10:**
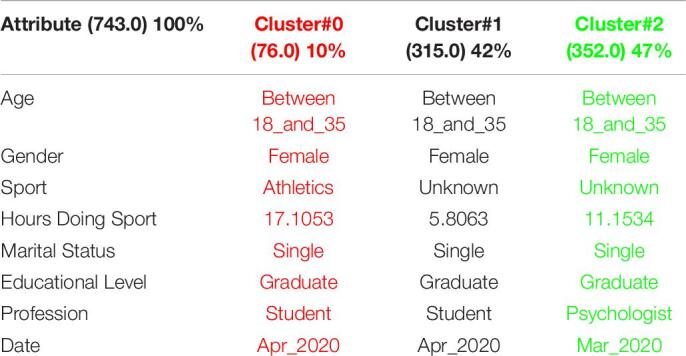
3 clusters IPED analysis for March to May 2020.

There is one important change about the gender of people taking the IPED survey. In 2019, about 50% of people were male and 50% female. However, in 2020, all of them were female. It is also noted in 2020 people is older and they have a higher education level, being graduated all of them.

**FIGURE 13:**
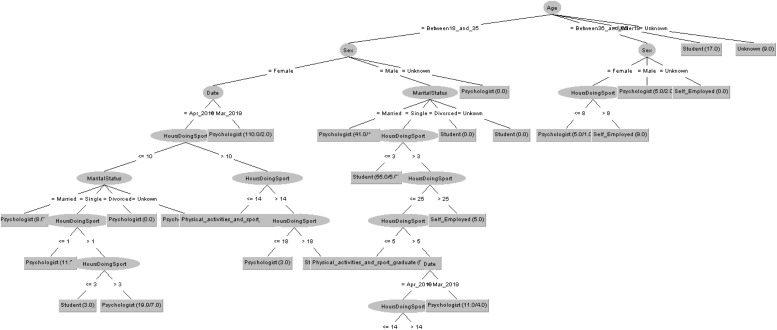
Profession of people taking the IPED questionnaire in 2019 decision tree.

**FIGURE 14:**
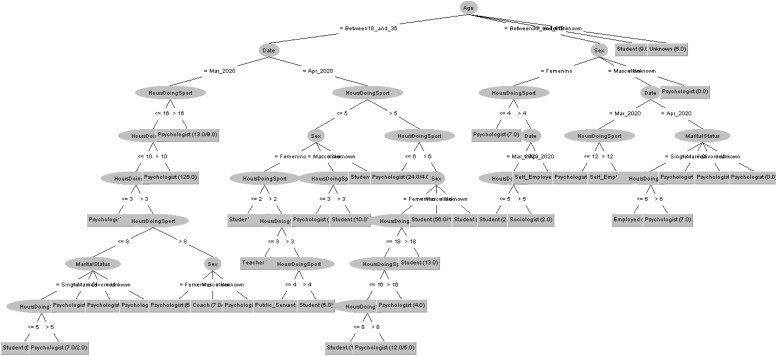
Profession of people taking the IPED questionnaire in 2020 decision tree.

In order to respond to the question raised before, Figures 13, 14 decision trees shows that most of people taking the IPED questionnaire are either Psychologist or students but there is not a clear pattern.

### Maslach Burnout Inventory (MBI)

Table 11 shows the 3 clusters got for RS questionnaire in 2019 and Table 12 shows the 2020 results. Red columns represent the less significate cluster attending to the number of elements belonging to the cluster and green columns represent the most significate one. Each cluster also shows the number of people belonging to it and the per cent it represents.

**TABLE 11:**
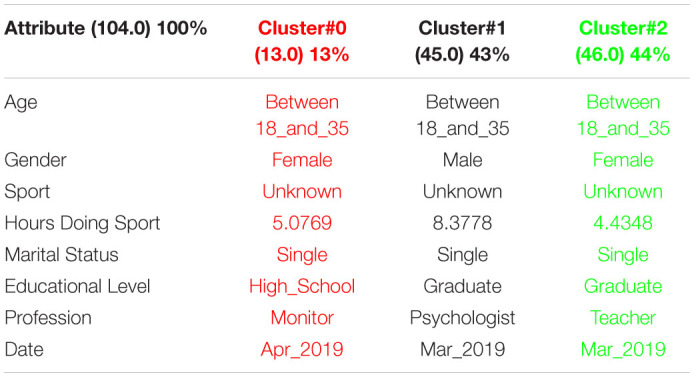
3 clusters MBI analysis for March to May 2019.

**TABLE 12:**
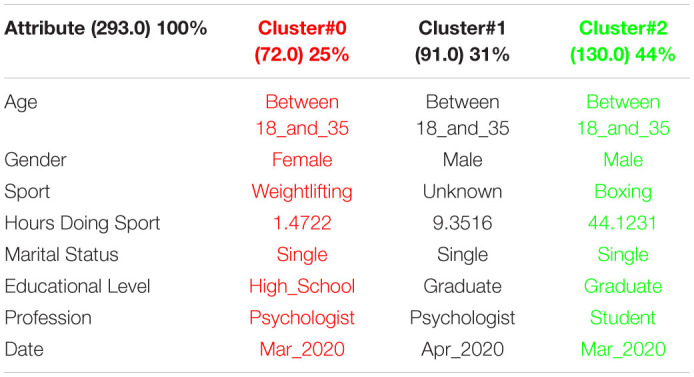
3 clusters MBI analysis for March to May 2020.

There is one important increase of males taking the MBI survey from 2019 to 2020. In 2019, about 43% of people were male and 57% female. However, in 2020, 74% of people were male and 26% female. This fact is due to many people doing boxing took the survey, so the typical person taking the MBI survey goes from a young female doing around 4 or 5 h of any sport in 2019 to a young male doing 44 h boxing in 2020.

**FIGURE 15:**
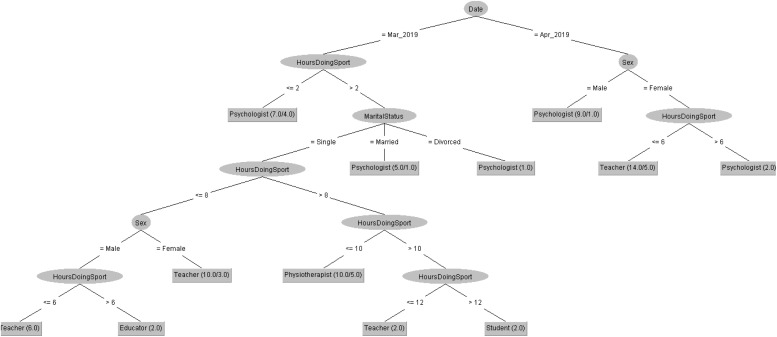
Profession of people taking the MBI questionnaire in 2019 decision tree.

**FIGURE 16:**
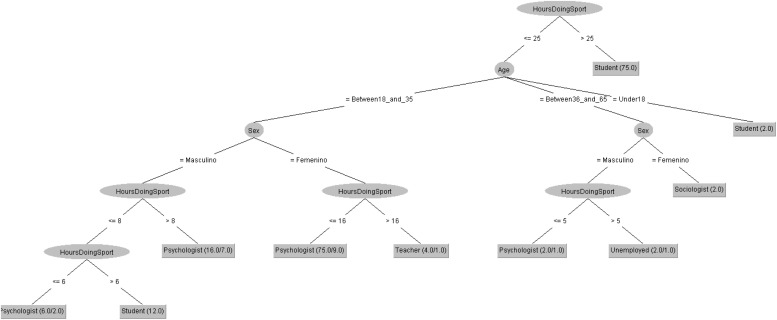
Profession of people taking the MBI questionnaire in 2020 decision tree.

In order to respond to the question raised before, Figures 15, 16 decision trees shows that most of people taking the MBI questionnaire are either Psychologist or students being date and hours doing sport in 2019 and age and hours doing sport in 2020 as main factors but there is not a clear pattern.

### Self-concept Form-5 (AF-5)

Table 13 shows the 3 clusters got for AF-5 questionnaire in 2019 and Table 14 shows the 2020 results. Red columns represent the less significate cluster attending to the number of elements belonging to the cluster and green columns represent the most significate one. Each cluster also shows the number of people belonging to it and the per cent it represents.

**TABLE 13:**
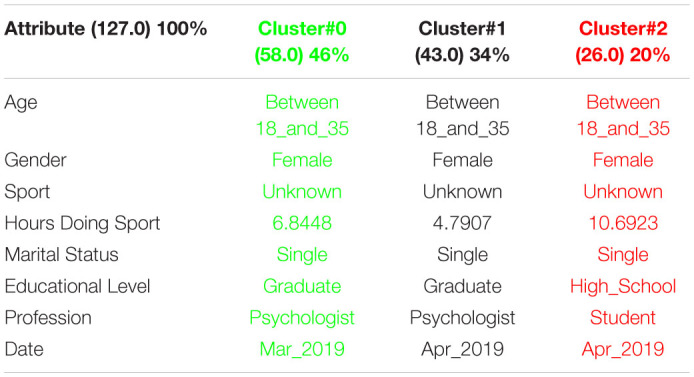
3 clusters AF5 analysis for March to May 2019.

**TABLE 14:**
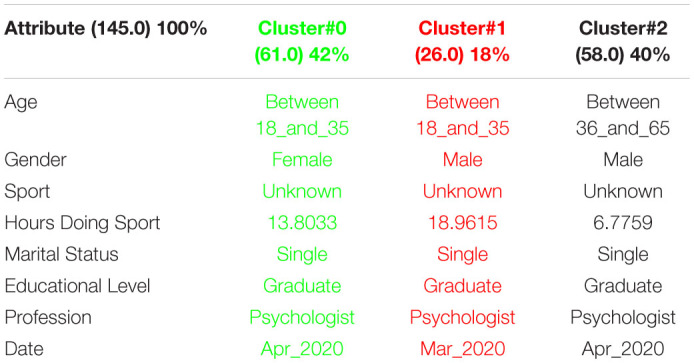
3 clusters AF5 analysis for March to May 2020.

It can be seen a significate fact in gender because in 2019 there is no cluster representing males, but in 2020 we found one cluster that represents to the 20% of the people taking the survey of males between 18 and 35 years old. We also can see that the number of hours doing sport have been increased in 2020 as well as the education level.

**FIGURE 17:**
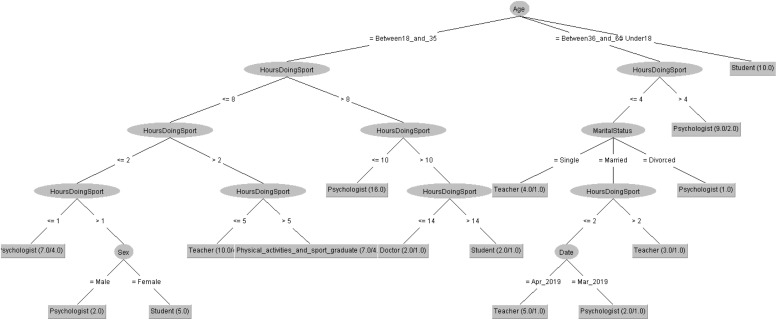
Profession of people taking the AF5 questionnaire in 2019 decision tree.

**FIGURE 18:**
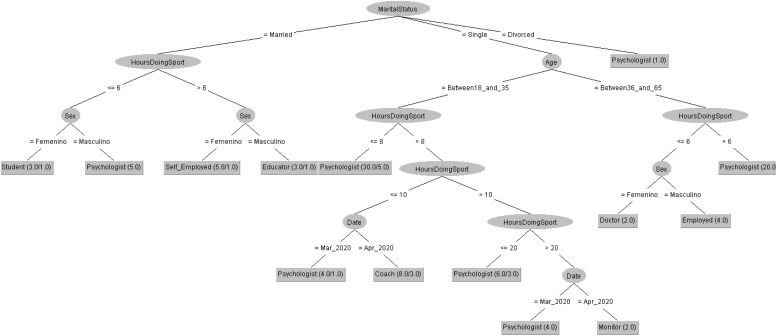
Profession of people taking the AF5 questionnaire in 2020 decision tree.

In order to respond to the question raised before, Figures 17, 18 decision trees shows that most of people taking the AF-5 questionnaire are either Psychologist or teachers being date and hours doing sport in 2019 and marital status, age and hours doing sport in 2020 as main factors but there is not a clear pattern.

## Discussion

The main objective was to determine if the COVID-19 Pandemic has changed the pattern of Menpas users. In order to check this, the following questionnaires taken by people all over the world, have been explored: Competitive State Anxiety Inventory-2 (CSAI-2), State Trait Anxiety Inventory (STAI), Profile of Mood State (POMS), Resilence Scale (RS), Sport Performance Psychological Inventory (IPED), Maslach Burnout Inventory (MBI) and Self-concept Form-5 (AF-5). First, a noticeable increase in the number of users has been observed, going from 11263 records from March the 1st to April the 30th in 2019 to 20627 records from March the 1st to April the 30th in 2020. Second, the pattern of MenPas users has also changed, indicating that there have been changes in the way the platform is used and in the type of user that MenPas has implemented.

The increase in the use of MenPas highlights how online platforms have been one of the ways in which many activities have been going on (Basilaia and Kvavadze, 2020; Belzunegui-Eraso and Erro-Garcés, 2020). The technological transition, possibly, has been accelerated due to this pandemic situation and generates a paradigm shift in the strategies to approach productive or formative procedures, etc., (Dong et al., 2020; Kousha and Thelwall, 2020). Educational and research activities are, among others, those that have caused a greater amount of data traffic through MenPas in recent years (e.g., Aragón et al., 2017; Reigal et al., 2019; González-Guirval et al., 2020). Therefore, these same factors could also be those that have caused a great increase in the use of the months of 2020 analyzed. In addition, this platform contains tools that are commonly used in the field of applied psychology for different issues (González-Ruiz et al., 2010, 2018), such as evaluations in athletes. This could be other reasons why its use has exploded.

For the CSAI-2, it was observed that the most representative cluster in 2019 was made up of people from 18 to 35 years old, female, single and psychologist. In 2020 some of these characteristics are maintained, although the most representative group is now made up of men aged between 36 and 65. For STAI, it was observed that the most representative cluster in 2019 was made up of people from 18 to 35 years old, female, single and psychologist. In 2020 the most representative cluster consisted of single male psychologists aged between 18 and 35 years. For POMS, something similar is also highlighted. The age range and marital status are maintained, although the most representative cluster goes from female psychologists in 2019 to male students in 2020. For RS it occurs as for STAI in characteristics such as age or marital status, although in this case the most representative cluster goes from men in 2019 to women in 2020.

For IPED there are no changes in the most representative cluster, although the least representative changes from male married psychologists in 2019 to single female students in 2020, all between 18 and 35 years old. For AF5 there are no changes in the most representative cluster either, but for the least. In 2019, they were single female students but in 2020 single male psychologists, all between 18 and 35 years old. For MBI, the most representative cluster changes. In 2019 it is formed by women between the ages of 18 and 35, single professors by profession. In 2020, it is made up of single men and students, aged between 18 and 35.

The results found do not allow obtaining a clear pattern of use. However, some important elements can be highlighted. First, there has been an increase in the number of users and changes in the characteristics of the users. This already constitutes a relevant fact and highlights changes in the way the platform is used. For example, the CSAI-2 has been used more by people between 36 and 65 years, which could indicate that a greater number of professionals in sports psychology have been able to use this instrument in their applied work (Belzunegui-Eraso and Erro-Garcés, 2020). In Spain the limitation on mobility was imposed during this time, but in other countries the restrictions have been different. For this reason, it is considered that in other countries MenPas could have been used as a way of having less personal contact, even while maintaining possible competitions for athletes. In addition, the CSAI contains a self-confidence scale, which could be used to monitor the athlete’s psychological state during confinement (Martens et al., 1990).

Secondly, it is seen in some questionnaires, such as POMS, IPED, AF5, or MBI, that students constitute the most representative group in 2020, without being in 2019, or that the least representative group ceases to be that of students. This constitutes perhaps the most relevant fact of this study, which shows that numerous educational institutions have been able to increase the use of this type of platform for the education of their students (Bao, 2020; Basilaia and Kvavadze, 2020; Belzunegui-Eraso and Erro-Garcés, 2020). These questionnaires are widely used in the field of psychology, both in education and in research (González et al., 2019; Chen et al., 2020; Ramírez-Siqueiros et al., 2020). Therefore, it is consistent to think that they have been used during this period to maintain this type of activities.

### Limitations and Future Works

Although there is a significant increase in the use of MenPas, there are aspects that are difficult to estimate. For example, in the months studied, not all countries have carried out the same measures in the face of the pandemic caused by SARS-CoV-2 (coronavirus 2019, COVID-19) because of the different evolution of the pandemic and how the virus has been spread over the world. Therefore, it would be interesting to assess the specific situations of confinement or alarm states in the different countries. In this way, it could be specified how each socio-political context affects the use of online technological resources. Likewise, it would be relevant to know the objective for which MenPas has been used. Although this can be relatively inferred, it is not precisely known. If we had that data, it could generate a more accurate profile of the users who use this resource and better understand how these instruments are used in each area but the main goal of this study is to get a significant knowledge about patterns in the use of on-line platforms when a socio-economic and environmental situation changes, so why users have used the platform is not so relevant to this study.

On the other hand, no data from similar platforms have been found because the known similar platforms like On-line Psychology Research,^4^ Social Psychology Network^5^ or Psicoactiva^6^ does not provide that kind of data. As soon as they appear, the results will be compared to assess the use that people make of this type of tools in a more precise way. Although changes are seen in user profiles, it is difficult to establish a unique pattern. Therefore, more data would be necessary to determine how each stratum of the population has changed their habits and to know what their needs may be in situations like this. We have been monitoring Menpas use after the study and the number of new users in 2020 (95) are around ten times more than 2019 (8) and 25 times more than 2018 (4). So, that means we have to analyze new data in the future checking use and patterns evolution.

## Conclusion

Data gathered in this study reveals a significant increase in data traffic and the number of MenPas users during the months of March and April 2020 due to the coronavirus pandemic. Furthermore, the characteristics of users have changed, highlighting changes in people’s habits. This fact shows that people change and modify their behavior and find a way to adapt and achieve their needs when there is a suddenly and hard change in the socio-economic and environmental situation where and when they live. However, more data is necessary to establish specific profiles and determine needs that can be addressed through this type of online platform.

## Data Availability Statement

The raw data supporting the conclusions of this article will be made available by the authors, without undue reservation.

## Author Contributions

AH-M, VM-S, RR, SG-R, JP-B, and JM-B participated in the study design and data collection, performed statistical analyses and contributed to the interpretation of the results, wrote the manuscript, approved the final manuscript as submitted, and reviewed and provided feedback to the manuscript. All authors made substantial contributions to the final manuscript.

## Conflict of Interest

The authors declare that the research was conducted in the absence of any commercial or financial relationships that could be construed as a potential conflict of interest. The reviewer CF declared a past collaboration with several of the authors (RR, AH-M, and VM-S) to the handling Editor.

## Footnotes

2 https://analytics.google.com

3 https://clustrmaps.com/site/19nkt

4 http://www.onlinepsychresearch.co.uk/

5 http://www.socialpsychology.org

6 http://www.psicoactiva.com
